# Biocontrol of *L. monocytogenes* with Selected Autochthonous Lactic Acid Bacteria in Raw Milk Soft-Ripened Cheese under Different Water Activity Conditions

**DOI:** 10.3390/foods13010172

**Published:** 2024-01-04

**Authors:** José M. Martín-Miguélez, Jurgen Robledo, Irene Martín, Cristina Castaño, Josué Delgado, Juan J. Córdoba

**Affiliations:** 1 Higiene y Seguridad Alimentaria, Instituto Universitario de Investigación de Carne y Productos Cárnicos (IProCar), Facultad de Veterinaria, Universidad de Extremadura, 10003 Cáceres, Spain; jmmm@unex.es (J.M.M.-M.); iremartint@unex.es (I.M.); cristinacs@unex.es (C.C.); jdperon@unex.es (J.D.); 2 Laboratorio Hidromante S.L., C. Isaac Peral, 15. Pol. Ind. Sepes, 10600 Plasencia, Spain; jurgen@hidromante.com

**Keywords:** *Lacticaseibacillus casei*, *L. monocytogenes* control, cheeses

## Abstract

The effect of selected autochthonous Lactic Acid Bacteria (LAB) against *Listeria monocytogenes* was evaluated in two elaborations of soft-ripened cheese performed under high and low relative humidity (RH) elaborations, to achieve a_w_ ranging from 0.97 to 0.94 in ripened cheeses. Two selected autochthonous strains of *Lacticaseibacillus casei* 31 and 116 were used. In each elaboration, 8 batches were physicochemically and microbiologically evaluated throughout the ripening process. The a_w_ and pH decreased during ripening to final values ranging from 0.944 to 0.972 a_w_ and 5.0 to 5.3 pH, respectively. LAB was the only microbial group that increased throughout the ripening in high and low RH elaborations. In batches that were uninoculated with LAB strains, *L. monocytogenes* was either maintained at the initial inoculation level or showed a slight reduction by the end of the ripening process. However, in LAB-inoculated batches in the two elaborations, steady decreases of *L. monocytogenes* were observed throughout maturation. *L. casei* 31 alone or in combination with strain 116 provoked reductions of 2 to 4 log CFU/g in *L. monocytogenes* over 60 days of ripening, which could be enough as a strategy for biocontrol to deal with the usual contamination by *L. monocytogenes* during cheese processing.

## 1. Introduction

*Listeria monocytogenes* is a ubiquitous Gram-positive bacterium usually transmitted to humans through food due to its wide distribution in food processing facilities, causing gastroenteritis, abortion, or even death, depending on the host’s susceptibility [1,2]. This pathogen is a persistent bacterium able to form biofilms on surfaces in food processing facilities and to adapt to stressful conditions, such as temperatures from −0.4 to 45 °C, 4.6–9.5 pH, and salt concentrations over 20%, making it a cause of public health and economic burden [3,4]. Its ability to survive under a wide range of physicochemical parameters, even after decontamination technologies have been applied, proves how listeriosis is a food safety hazard, with ready-to-eat (RTE) foods, such as soft-ripened cheeses, being the most important vehicle of transmission to humans [5,6].

Most cheeses present physicochemical characteristics that allow *L. monocytogenes* to survive and grow, causing listeriosis outbreaks regardless of the country of study [7,8,9]. The hazard of the presence of *L. monocytogenes* in soft-ripened cheeses made with raw milk is higher than in those whose elaboration includes a pasteurization step, due to the possible presence of this pathogen in raw milk [10,11].

Microbial control has traditionally been achieved in this kind of soft-ripened cheese by salting, acidification, drying, and chemical preservation [12]. Nowadays, consumers concern about the presence of chemical compounds among food ingredients, have promoted the development of more sustainable preservation methods, such as high hydrostatic pressure, cold plasma, pulsed light, ultrasound, bioactive films, natural antimicrobial agents such as plant extracts, or biocontrol by microbial protective cultures [13,14]. The last method is recommended due to its low environmental impact and easiness of application [15].

“Torta del Casar” is one of the traditional soft-ripened cheeses made from raw milk, for which Protected Designations of Origin (P.D.O.) regulations forbid any practice or addition of substances external to the product itself; yet, at the same time, its physicochemical characteristics may allow the development of pathogenic microorganisms such as *L. monocytogenes* [16]. In addition, in the elaboration of this kind of soft-ripened cheese that is usually made under traditional conditions, the a_w_ can vary between 0.98 and 0.94 throughout the ripening, resulting in a_w_ values ranging from 0.97 to 0.94 in the final product due to differences in relative humidity (RH) during the traditional process, which usually varies from 90% to 70% RH. These differences can be critical for the persistence and even growth of *L. monocytogenes*. Biocontrol with selected protective cultures of lactic acid bacteria (LAB) has been shown to be an effective method for reducing this pathogenic bacterium in “Torta del Casar” soft-ripened cheese [17]. In other P.D.O. cheeses from Europe, the use of LAB as protective cultures has proved to be an efficient strategy to achieve a reduction of around 2 log CFU/g of *L. monocytogenes* [18,19]. However, the efficiency in the reduction of *L. monocytogenes* with selected LAB strains has always been demonstrated in cheeses reaching a_w_ values below 0.94 at the end of processing [7]. Nevertheless, the efficiency of selected autochthonous LAB against *L. monocytogenes* in soft-ripened cheese with higher a_w_ values (0.97 to 0.94) in the final product has not been evaluated yet, even though these variations can be found in traditional soft-ripened cheeses and can be critical to controlling *L. monocytogenes*.

This work aimed to evaluate selected LAB for the biocontrol of *L. monocytogenes* in the traditional soft-ripened cheese “Torta del Casar” under different relative humidity conditions during ripening. In addition, the physicochemical and microbiological modifications in the LAB-inoculated cheeses throughout the ripening process were evaluated. 

## 2. Materials and Methods

### 2.1. Microbial Cultures

*L. monocytogenes* strain S7-2 (serotype 4b) belonging to the National Institute of Agricultural and Food Research and Technology (INIA) Culture Collection (Madrid, Spain) was utilized throughout the experiment carried out. The LAB strains used, “31” and ”116” (*Lacticaseibacillus casei*), were isolated from “Torta del Casar” cheese and selected by their anti-*L. monocytogenes* activity in cheese-based agar in vitro models [20]. *L. monocytogenes* and LAB were sub-cultured twice into 10 mL of brain heart infusion (BHI; Conda Pronadisa, Madrid, Spain) and Man Rogosa Sharpe (MRS; Conda Pronadisa), respectively, from frozen stock cultures (−80 °C) of the same media containing 20% (*w*/*v*) of glycerol (Thermo Fisher Scientific, Waltham, MA, USA).

At the end of the incubation period, microbial cells were centrifugated at 10,621 g for 10 min; the supernatants were discarded; and cells were dissolved in phosphate buffered saline (PBS; 0.32 g of sodium dihydrogen phosphate (Scharlau Chemie S.A., Barcelona, Spain), 1.09 g of disodium hydrogen phosphate (Scharlau Chemie S.A.), 9 g of NaCl (Thermo Fisher Scientific), 1 L of distilled water), achieving a concentration of ≈11 log CFU/mL for LAB and ≈4–5 log CFU/mL in the case of *L. monocytogenes*. Serial dilutions were carried out to verify the inoculation load; LAB was incubated on MRS agar at 30 °C and *L. monocytogenes* on Agar *Listeria* Ottaviani and Agosti (ALOA; Conda Pronadisa) at 37 °C for 48 h. MRS agar and ALOA are selective media for LAB and *L. monocytogenes,* respectively. Moreover, the initial counts (CFU/g) were assessed at the beginning of the maturation time.

### 2.2. Preparation of “Torta del Casar” Cheeses

“Torta del Casar” cheeses were elaborated in the facilities of a P.D.O. manufacturer in Casar de Cáceres (Extremadura region, Spain) to bring this study close to the commercial scale. Two separate elaborations were performed at two different times of the year (winter and summer) to evaluate the heterogeneity in relative humidity conditions during ripening and consequently in the a_w_ of the product throughout the processing of an artisanal product such as “Torta del Casar” and to study the use of LAB in several scenarios. In each production, four batches were produced: A (control without LAB added), B (with LAB 116), C (with LAB 31), and D (LAB 116 + 31). The material utilized during processing was the same material that the facility uses to elaborate their commercial products. Fifty liters of raw ewe milk collected from several farms were used for each batch with the addition of 10 mL of PBS with a LAB concentration of 11 log CFU/mL to reach 6 log CFU/mL in the milk to be processed. Thirty fresh pieces of approximately 500 g of cheese from each of the above-mentioned batches were made under the employees’ supervision, following their usual routine for “Torta del Casar” which involves cheese curdling for 1 h at 28 °C with vegetable coagulant obtained from flowers of *Cynara cardunculus* L., pressing for 1 h, and salting in brine for 2 h. Hygienic measures were taken to avoid contamination between batches following the industry-established protocol and using the facility’s disinfection materials. 

The elaborated fresh cheeses were transported on the same day of production to the Faculty of Veterinary of the University of Extremadura under refrigeration conditions (3 °C ± 0.5) for inoculation with *L. monocytogenes* and further ripening. Cheeses from every batch were inoculated the same day in a laminar flow cabinet (Azbil Telstar Technologies S.L.U., Barcelona, Spain) with a 4 log CFU/mL PBS concentration of *L. monocytogenes* in the center of the product (in a cube of 16 cm^2^ of surface and 6 cm deep [the entire volume of fresh cheese], and ≈100 g of weight) according to Martín et al. [17]. A concentration of 4–5 log CFU/g was intended to be achieved on the initial day of ripening. The four original batches (A, B, C, and D) were subdivided into batches without inoculation by *L. monocytogenes* (A, B, C, and D) for physicochemical analyses and four additional batches inoculated with *L. monocytogenes*: E (from batch A only inoculated with *L. monocytogenes*), F (from batch B inoculated with LAB 116 + *L. monocytogenes*), G (from batch C inoculated with LAB 31 + *L. monocytogenes*), H (from batch D inoculated with LAB 116 + 31 + *L. monocytogenes*). Fifteen cheeses belonging to each one of the 8 batches were ripened for 60 days (minimum ripening time of processing according to P.D.O. regulations) at the pilot plant of the Faculty of Veterinary (Extremadura region, Spain).

The conditions followed by maturation in the two processes were slightly different in terms of relative humidity (RH), following the usual fluctuations of this parameter in the traditional ripening of this kind of soft-ripened cheese. Thus, maturation 1 (named Low RH) was processed as follows: 35 days at 6 °C and 90% RH, 10 days at 8 °C and 70% RH, 10 days at 9 °C and 70% RH, and 5 days at 10 °C and 70% RH. The conditions for maturation 2 (named High RH) were: 35 days at 6 °C and 90% RH, 10 days at 8 °C and 80% RH, 10 days at 9 °C and 80% RH, and 5 days at 10 °C and 80% RH.

In each maturation, 3 cheeses of each batch (A, B, C, D, E, F, G, and H) were taken for physicochemical and microbiological analyses in the different sampling days (0, 15, 30, and 60 days of ripening). Samples were taken in a laminar flow cabinet (Azbil Telstar Technologies) from the central cube of the cheeses, with 16 cm^2^ of surface, 6 cm depth, and ≈100 g in weight. This is the part of the cheese that was inoculated with *L. monocytogenes* as previously described.

### 2.3. Microbiological Analysis

Microbiological analyses were performed for each elaboration on days 0, 15, 30, and 60, by taking the central cube comprising 16 cm^2^ (except for the outer layers) and homogenizing the cheese in Stomacher homogenizer bags (Interscience, Saint-Germain-en-Laye, France). Then, 25 g of cheese from batches A, B, C, and D was removed to confirm the absence of natural contamination of *L. monocytogenes* according to the International Organization for Standardization (ISO) 11290-1 standard [21]. In addition, 10 g of each homogenized cheese from every batch were analyzed with 90 mL of 1% (*w*/*v*) peptone water (Conda Pronadisa); subsequently, decimal dilutions of peptone water were prepared, and agar plates from different media according to the microbial group studied were used. Plate count agar (PCA; Conda Pronadisa), mannitol salt agar (MSA; Conda Pronadisa), and MRS were utilized to sample total viable counts, Gram-positive catalase-positive cocci (GC+), and LAB, respectively, after incubation at 30 °C for 72 h, except in the case of MRS, which was incubated for 48 h under microaerophilic conditions using a plastic bag to provide an oxygen-deprived environment. Malt Extract Agar (MEA; 20 g of malt extract (Conda Pronadisa), 20 g of glucose (Labbox Labware S.L., Barcelona, Spain), 1 g of bacteriological peptone (Conda Pronadisa), 20 g of bacteriological agar (Conda Pronadisa), 1 L of distilled water) was used to evaluate yeasts after being incubated at 25 °C for 48 h. Violet red bile glucose agar (VRBG; Conda Pronadisa) was incubated at 37 °C for 24 h to determine *Enterobacteriaceae*. *L. monocytogenes* quantification was carried out just in batches E, F, G, and H on ALOA, which allowed the differentiation of *L. monocytogenes* from other *Listeria* spp. after 48 h at 37 °C, by their development of green color colonies surrounded by the characteristic opaque halo. The counts were performed in plates with more than 10 colonies and less than 300 [22], and they were expressed at CFU/g.

### 2.4. Physicochemical Analysis

Physicochemical analyses were conducted according to the Association of Official Agricultural Chemists [23]. They were performed for each production on days 0, 15, 30, and 60 for batches A, B, C, and D, by taking the homogenized cheese used for microbial analyses. The samples were homogenized in ultrapure water to be measured with a pH meter (Hach Lange Spain S.L.U., Barcelona, Spain) previously calibrated with standard solutions (Scharlau Chemie S.A.). The a_w_ was measured with a Novasina Lab Master Water activity meter model a_w_ SPRINT-TH 300 (Novasina AG, Lachen, Switzerland). Moisture content was calculated through the dehydration of samples at 105 °C with the addition of previously dehydrated washed sea sand (Scharlau Chemie S.A.).

### 2.5. Characterization of LAB Strains by Pulsed-Field Gel Electrophoresis Typing

To evaluate the implantation of LAB 116 and 31 during ripening, LAB-inoculated batches sampled at the end of the ripening time and inoculated onto MRS plates, grew on this medium and were selected from each production. Fifty percent of the colonies on each MRS plate were isolated and inoculated into MRS broth to get a higher cell concentration after incubation at 30 °C for 48 h. LAB 116 and 31 were also inoculated into MRS broth from their frozen stock cultures and incubated under the same conditions to be used as standards. LAB strains were characterized by pulsed-field gel electrophoresis (PFGE) according to Martín et al. [20].

### 2.6. Statistical Analysis

Different statistical analyses were performed using IBM SPSS Statistics v.22 (IBM Co., New York, NY, USA, EEUU), segmenting by batch and analyzing days as the dependent variable, and vice versa. Shapiro–Wilk and Levene’s tests were performed to evaluate normality distribution and homogeneity of variances, respectively. Normal and homogeneous variables were analyzed with the HSD Tukey test; the T3 Dunnet was used to study the statistical differences between normal and non-homogeneous variables; and the Kruskal-Wallis test was carried out on non-normal variables.

## 3. Results

### 3.1. Physicochemical Changes during Ripening

The evolution of the moisture content, a_w_, and pH throughout the ripening process in the different batches and the two elaborations are shown in Table 1. The moisture content in batches produced under Low RH conditions decreased in all the analyzed batches from initial values on day 0 of ripening, which ranged from 57–55%, to 32–39% by the end of maturation. However, in the High RH elaboration, the decrease in humidity content only reached 43–45% in all batches by the end of the processing. At days 30 and 60 of ripening, the humidity content was significantly (*p* ≤ 0.05) higher for High RH than for the Low RH elaboration for all the analyzed batches (Table 1). 

The a_w_ values decreased during ripening to reach values that ranged from 0.944–0.962 in finished cheeses under Low RH maturation and values ranging from 0.962 to 0.972 in the final cheeses under High RH elaboration. Only in A and B batches, the a_w_ values were significantly (*p* ≤ 0.05) lower for Low RH than for the High RH elaboration (Table 1).

The pH of cheeses decreased during the first 30 days of ripening from initial values around 7 to levels below 5 in all batches of the Low RH elaboration and below 5.4 in all batches of the High RH maturation. From day 30 to 60 of ripening, the pH slightly increased (*p* ≤ 0.05) for the Low RH elaboration, reaching values around 5.0–5.1, but remained around 5.3 in High RH ripening (Table 1). However, no significant differences (*p* ≤ 0.05) were found in the final pH values between batches from each elaboration except for batch D, which displayed higher (*p* ≤ 0.05) values for the High RH elaboration than for the Low RH elaboration.

### 3.2. Microbiological Changes during Ripening

The levels of all analyzed microbiological groups, except *L. monocytogenes*, throughout the ripening process in Low and High RH elaborations are displayed in Table 2. Total viable counts (TVC) increased (*p* ≤ 0.05) from values around 6 or 7 log CFU/g at day 0 to levels close to 8 or 9 log CFU/g at day 15 of ripening in all batches of both maturations. In the remaining days of ripening, the levels of TVC remained at the same level or even decreased in all batches of both elaborations, except for B, F, and H batches, which showed slight increases (*p* ≤ 0.05) to levels ranging from 9.18 to 9.52 log CFU/g (Table 1). By the end of ripening, there were no significant differences between batches of either elaboration, but significantly (*p* ≤ 0.05) higher TVC levels were found in C, E, F, G, and H batches of the High RH elaboration.

*Enterobacteriaceae* counts increased (*p* ≤ 0.05) in the first 15 days of elaboration to reach levels ranging from 7 to 8 log CFU/g in all batches of both Low and High RH elaborations (Table 1). Then, levels of *Enterobacteriaceae* decreased at day 60 in both elaborations. In Low RH maturation, levels of *Enterobacteriaceae* at days 15, 30, and 60 of ripening were significantly higher in A and E batches (without LAB) than in most of the LAB-inoculated ones (Table 2).

Gram-positive catalase-positive cocci (GC+) showed levels generally ranging from 4 to 6 log CFU/g in all batches of the two elaborations, showing increases in the first 15 days of processing (Table 2). From day 15 to the end of ripening, there were no increases in this microbial group, and even some decreases were observed. At the end of ripening, counts of GC+ were lower (*p* ≤ 0.05) than those found on the rest of the days of processing for most of the batches. The levels of GC+ at day 60 of maturation were significantly (*p* ≤ 0.05) lower in all batches of High RH elaboration in comparison with Low RH maturation (Table 2).

LAB levels increased (*p* ≤ 0.05) in most batches of Low and High RH elaborations at days 15, 30, and 60 of ripening (Table 2). At the end of ripening, the highest counts were always found in LAB-inoculated batches (B, C, D, F, G, and H) of both Low and HR elaborations. In these batches, levels of LAB were higher than 7 log CFU/g throughout the ripening period. At day 60 of maturation, LAB counts of the High RH maturation showed significantly higher levels in D, E, F, and H batches than in the same batches of the Low RH elaboration.

Yeast counts increased (*p* ≤ 0.05) at day 15 in all batches of the two elaborations (Table 2). In the next days of ripening, steady increases until day 60 were found only for the High RH elaboration. At the end of ripening, levels of yeasts were higher than 8 log CFU/g in all batches and elaborations. 

Levels of *L. monocytogenes* throughout the ripening are displayed in Table 3 for both elaborations (Low and High RH). In the E batch (uninoculated with LAB), during the first 30 days of ripening, *L. monocytogenes* showed a significant (*p* ≤ 0.05) increase under Low RH maturation, while under High RH maturation, levels of *L. monocytogenes* remained at inoculation levels in the first 15 days of ripening and decreased to less than 1 log CFU/g at day 30 of ripening. In any case, a slight decrease of 1 log CFU/g of *L. monocytogenes* since the initial day was only observed at the end of ripening in batch E of the High RH elaboration (Table 3). On the contrary, in the LAB-inoculated batches of both Low and High RH elaborations, continuous reductions of *L. monocytogenes* counts compared to initial inoculation levels were observed throughout the ripening time. At the end of ripening, the reduction (*p* ≤ 0.05) of *L. monocytogenes* in the LAB-inoculated batches was around 1.5–2 log CFU/g in Low RH batches and 3.5–4 log CFU/g in High RH ones (Table 3). The highest reductions of this pathogen in the two elaborations were observed in batch G (inoculated with LAB 31) and especially in batch H (inoculated with LAB 116 and 31). 

### 3.3. Characterization of LAB Strains by Pulsed-Field Gel Electrophoresis Typing

The PFGE profiles obtained from the LAB isolates of inoculated batches were identified by comparison with the standard LAB 116 and 31 PFGE profiles. The analyzed LAB isolates showed similar restriction patterns to the original inoculated 116 and 31 bacteria on the final day of ripening. The 116 LAB-inoculated cheeses showed a 75% of similarities and the 31 LAB-inoculated a 66%.

## 4. Discussion

Raw milk soft-ripened cheeses represent a consumer hazard as they have not undergone a pasteurization process [16] and may allow the development of *L. monocytogenes* during processing and commercialization due to their physicochemical characteristics. LAB have been proposed as an effective strategy to inhibit the growth or even decrease levels of *L. monocytogenes* in ripened products [15]. However, there is doubt about whether LAB can control this pathogenic bacterium under conditions of soft-ripened cheese made with raw milk, in which a_w_ during ripening and in the final product is, in most cases, in the range of 0.94–0.97.

In the present work, two different elaborations of cheeses were carried out to evaluate the efficiency of selected autochthonous LAB in controlling *L. monocytogenes* over a wide range of moisture content and a_w_, including values of the latter at which control of this pathogenic bacterium can be more challenging. The two maturations allowed cheeses with different moisture content in the range of 32 to 46%, with higher values being observed for High HR maturation than for Low HR maturation during ripening and in final products. In all cases, the evolution of moisture content throughout the ripening period and the final content of moisture were in the reported range for this kind of cheese [24,25,26]. Although the a_w_ decreased in both elaborations as expected, the decrease was slower under High RH than under Low RH maturation. This allowed us to obtain a wide range of a_w_ values (from 0.94 to 0.97) in the cheeses, throughout the ripening process and in the final products. Higher values were observed in High RH than in the Low RH elaboration. The a_w_ values obtained were also in the range reported for this kind of cheese [24,25,26] and were not sufficient to control *L. monocytogenes* since they never reached values below 0.92 [27].

The pH decreased during the first 30 days of ripening in the two maturations followed by values that ranged between 4.82 and 5.36 but, in any case, reached values ≤4.4, which are reported as being able to inhibit *L. monocytogenes* by themselves [27]. From day 30 until day 60 of ripening, pH increased slightly until reaching final values of 5.0–5.3 in the two elaborations, probably due to the accumulation of products derived from the proteolysis [17]. The final pH values cannot control *L. monocytogenes* but, in combination with a_w_ (a_w_ ≤ 0.94 and pH ≤ 5.4), would inhibit this pathogenic bacterium [27], although these conditions were not found in any of the elaborations and batches analyzed. 

The differences in moisture content, a_w_, and pH between batches throughout the ripening process are mainly attributed to the different conditions of relative humidity during ripening and not to the LAB inoculation since there were no consistent differences in the above physicochemical parameters between LAB-inoculated and uninoculated batches. Thus, the addition of selected autochthonous LAB strains 116 and 31 did not provoke any modification of physicochemical parameters that influence the sensorial characteristics of soft-ripened cheeses that have been reported in previous works [17].

TVC, *Enterobacteriaceae,* and GC+ only showed significant increases in all analyzed batches in the first 15 days of the two elaborations studied. Similar increases have also been reported at the beginning of the maturation of traditional soft-ripened cheeses such as “Torta del Casar” and “Queso de la Serena” when a_w_ is above 0.96 [24]. This could also explain the higher counts of TVC found under High RH maturation, in which the moisture content and a_w_ were higher [25]. Levels over 7 log CFU/g of *Enterobacteriaceae* have also been observed in “Torta del Casar” cheeses in the second week of ripening, with an increase close to 2 log CFU/g relative to initial levels [24]. This microbial group has also been found in high numbers in raw milk soft-ripened cheeses at the end of maturation and has been reported to contribute to flavor development [26,28]. GC+ levels were always lower than 7 log CFU/g in all batches and elaborations analyzed. Remarkably, GC+ displayed levels around 6 log CFU/g with a moisture content between 30–40% under Low RH maturation and around 3.5–4.5 log CFU/g with a moisture content close to 45% under High RH elaboration. The GC+ usually found during the ripening of soft-ripened cheeses are more adapted to the values of a_w_ found under Low RH elaboration [17]. 

LAB and yeasts were the only microbial groups that showed increases in most batches at days 15, 30, and 60 of ripening of both elaborations; but in the case of yeasts, these increases were only observed under High RH maturation. Changes in moisture and a_w_ reduction over the course of ripening do not appear to affect the growth of either microbial group [17], except under Low RH maturation, in which the growth of yeasts seems to be more limited in comparison with High RH elaboration. It is worth noting that the LAB reached levels above 7 log CFU/g throughout the ripening period, and by the end of the processing, there were always higher LAB concentrations in the inoculated batches (B, C, D, F, G, and H) of both Low and High HR elaborations. However, inoculation by selected autochthonous LAB was not reflected in a greater decrease in pH in the LAB-inoculated batches, probably due to *L. casei* belonging to heterofermentative LAB, which can be positive for maintaining the sensorial characteristics of ripened cheeses. No negative effects have been described in soft-ripened cheeses matured with similar levels of LAB [29,30].

PFGE analysis of the LAB strains in the final products confirmed the correct implantation of *L. casei* 116 and *L. casei* 31 LAB in their respective batches, since these strains were the majority found in the corresponding ripened cheeses, which also indicates that these strains were present throughout the ripening time. 

**Figure 1:**
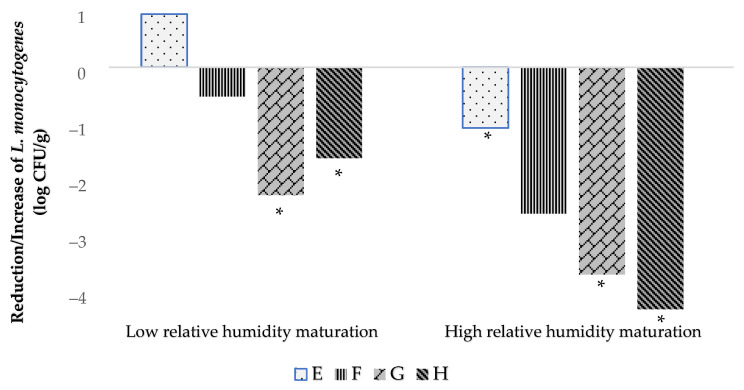
Evolution of *L. monocytogenes* counts during 60 days of ripening for low and high RH elaborations. E (inoculated with *L. monocytogenes*), F (inoculated with *L. monocytogenes* and LAB 116), G (inoculated with *L. monocytogenes* and LAB 31) and H (inoculated with *L. monocytogenes*, LAB 116 and 31). Values are expressed as mean and standard deviation in box and whisker plots. Values with an asterisk as a superscript (*) indicate significant differences (*p* ≤ 0.05) with day 0 of the same elaboration.

Regarding *L. monocytogenes*, the levels of this pathogen in the E batch (uninoculated with LAB) increased or were maintained at initial inoculation levels during the first 15 days of ripening. This can be explained because the conditions of a_w_ (around 0.98) and pH (between 5.8–5.3) do not inhibit *L. monocytogenes* and even promote its growth [31]. During the second half of maturation (from day 30 to 60) of the E batch, this pathogenic bacterium either remained at the inoculation level or showed a slight reduction of about 1 log CFU/g, probably due to the cheese values of a_w_ and pH (a_w_ ≤ 0.94 and pH ≤ 5.4) that do not favor the growth of *L. monocytogenes* [27]. However, in LAB-inoculated batches (F, G, and H) of both Low and High RH elaborations, continuous reductions of *L. monocytogenes* throughout the ripening time were observed, even though, in the first days of ripening, the a_w_ and pH conditions could promote growth of this pathogen [31]. Complete maturation of LAB-inoculated cheeses was able to reduce *L. monocytogenes* to the range of 1.51 to 4.07 log CFU/g (Figure 1).

The reduction in *L. monocytogenes* found in LAB 116- and 31-inoculated batches contrasts with that of batches not inoculated with LAB, in which this microbial group from contamination, mainly from raw milk, were at similar levels to the inoculated batches during ripening but in which the reduction of this pathogen was very low or absent. Thus, the inoculated LAB 116 and 31 exerted an antagonistic effect against *L. monocytogenes*, probably due to competition for nutrients and the production of compounds like bacteriocins that have been reported for *L. casei* and for the 116 strain used in the present work [32,33,34]. It deserves to be highlighted that LAB 31 alone or in combination with strain 116 provoke reductions of *L. monocytogenes* from 2 to 4 log CFU/g over 60 days of ripening (Figure 1). Several LAB strains of dairy origin have achieved reduction of *L. monocytogenes* in different types of cheeses elaborated in Brazil [35,36], Italy [37,38], and Spain [17]. In addition, reductions of about 1 log CFU/g of *L. monocytogenes* in soft-ripened cheese “Torta del Casar” inoculated with LAB have been previously reported [17]. However, in the present work, it is demonstrated that by using selected autochthonous LAB in soft-ripened cheeses, reductions of *L. monocytogenes* of up to 4 log CFU/g can be achieved during ripening under various a_w_ values, including those that promote the growth of this pathogenic bacterium. This is of utmost importance to ensure microbial safety concerning *L. monocytogenes* in this kind of soft-ripened cheese, in which the conditions of a_w_ and pH during part of the ripening time and in the final products are usually above those (a_w_ ≤ 0.94 and pH ≤ 5.4) that do not allow growth of this pathogenic bacterium [27]. Even under these conditions, the utilization of selected autochthonous LAB guarantees the elimination of *L. monocytogenes*. The possible contamination of this bacterium could reach the product through the raw milk, equipment, utensils, or food handlers, usually at levels lower than 2 log CFU/g [39]. This is of great relevance to minimizing the risk of listeriosis associated with the consumption of soft-ripened cheeses and to avoid non-compliance with the microbiological criteria of RTE foods in industry and throughout their shelf-life in the EU [27]. In addition, the presence of selected LAB strains could help to control the microbial contamination of raw milk soft-ripened cheeses [17,40]. In the present work, the levels of *Enterobacteriaceae* during ripening under Low RH maturation were significantly lower in the LAB-inoculated batches. 

Thus, the utilization of selected autochthonous LAB strains could be a good and sustainable strategy for traditional cheese industries to improve the safety of soft-ripened cheeses without affecting their physicochemical characteristics. However, the present study has applied certain selected autochthonous LAB to one kind of soft-ripened cheese, “Torta del Casar”, and further studies must be performed with more selected autochthonous LAB strains obtained from other kinds of cheeses to expand the application of LAB for the biocontrol of *L. monocytogenes* in raw milk cheeses. 

## 5. Conclusions

This work demonstrates that the utilization of selected autochthonous LAB as protective cultures in soft-ripened cheeses provoked reductions of *L. monocytogenes* of 1.5–4 log CFU/g during ripening under different conditions of a_w_, including those that promote the growth of this pathogenic bacterium, without affecting the physicochemical characteristics of the final products. The combined utilization of *L. casei* 31 and *L. casei* 116 caused the most consistent reduction of *L. monocytogenes* counts, by about 4 log CFU/g, over the course of the industrial ripening process, independently of the physicochemical characteristics of soft-ripened cheeses, which are proved to be variable according to the artisanal elaboration of the product. The reduction achieved by the mix of the above LAB may be enough to deal with the natural contamination of *L. monocytogenes*. This biocontrol strategy could be included in the HACCP framework as a preventive measure, to reinforce the high safety standards required for artisanal soft-ripened cheeses made with raw milk.

## 6. Patents

Martín, I., Rodríguez, A and Córdoba, J.J, inventor 2021. The new strain of *Lacticaseibacillus casei* 116 with antagonist effect against *Listeria monocytogenes* to be used as protective culture in ripened cheese. No. P202131120.

## Figures and Tables

**Table 1:** Evolution of moisture content (%), water activity (a_w_), and pH for low and high relative humidity soft-ripened cheese elaborations.

	Batches	Days of Ripening			
		0	15	30	60
1. Low relativity humidity maturation					
Moisture content (%)	A	55.34 ± 0.99 *^b4^	50.75 ± 2.18 ^3^	40.24 ± 0.70 *^2^	32.77 ± 1.05 *^a1^
	B	52.37 ± 1.22 *^a3^	48.99 ± 2.87 ^3^	40.14 ± 0.69 *^2^	33.75 ± 0.65 *^a1^
	C	57.85 ± 1.25 ^c23^	47.23 ± 1.25 *^3^	43.24 ± 0.72 *^2^	39.35 ± 0.22 *^b1^
	D	55.12 ± 0.78 ^b4^	48.65 ± 2.57 *^3^	38.73 ± 1.83 *^2^	33.61 ± 1.11 *^a1^
a_w_	A	0.980 ± 0.002 ^2^	0.980 ± 0.002 ^b3^	0.949 ± 0.002 *^1^	0.949 ± 0.003 *^a1^
	B	0.977 ± 0.001 *^3^	0.977 ± 0.001 ^ab4^	0.951 ± 0.004 *^2^	0.944 ± 0.001 *^ab1^
	C	0.982 ± 0.005 ^2^	0.982 ± 0.005 ^ab3^	0.950 ± 0.007 *^1^	0.962 ± 0.002 ^b1^
	D	0.979 ± 0.001 ^2^	0.981 ± 0.006 ^a2^	0.942 ± 0.005 *^1^	0.952 ± 0.010 ^ab1^
pH	A	6.93 ± 0.11 *^b2^	5.33 ± 0.29 ^1^	4.95 ± 0.13 *^1^	5.16 ± 0.04 ^1^
	B	6.69 ± 0.05 *^a3^	5.27 ± 0.02 ^2^	4.86 ± 0.04 *^1^	5.15 ± 0.23 ^12^
	C	6.61 ± 0.12 ^a2^	5.21 ± 0.10 ^1^	4.82 ± 0.25 ^1^	5.18 ± 0.24 ^1^
	D	6.65 ± 0.04 ^a2^	5.31 ± 0.22 ^1^	4.94 ± 0.10 *^1^	4.99 ± 0.03 *^1^
2. High relative humidity maturation					
Moisture content (%)	A	56.96 ± 0.44 *^3^	50.02 ± 0.77 ^a2^	51.80 ± 1.34 *^2^	43.25 ± 1.10 *^a1^
	B	56.18 ± 1.45 *^3^	51.47 ± 0.90 ^a2^	52.72 ± 0.91 *^23^	45.78 ± 0.23 *^a1^
	C	58.39 ± 1.11 ^3^	51.47 ± 0.66 *^a2^	54.14 ± 1.93 *^123^	47.78 ± 0.66 *^a1^
	D	56.49 ± 1.14 ^2^	53.90 ± 1.27 *^b2^	53.69 ± 1.28 *^2^	45.98 ± 1.03 *^b1^
a_w_	A	0.976 ± 0.024 ^23^	0.981 ± 0.004 *^3^	0.974 ± 0.004 *^2^	0.963 ± 0.010 *^b1^
	B	0.982 ± 0.004 *^2^	0.976 ± 0.005 *^2^	0.975 ± 0.007 *^12^	0.962 ± 0.005 *^ab1^
	C	0.980 ± 0.012 ^123^	0.982 ± 0.003 *^3^	0.976 ± 0.001 *^2^	0.967 ± 0.007 ^ab1^
	D	0.979 ± 0.001 ^2^	0.983 ± 0.001 ^2^	0.974 ± 0.001 *^1^	0.972 ± 0.008 ^b1^
pH	A	7.09 ± 0.29 *^bc2^	5.68 ± 0.25 ^2^	5.36 ± 0.28 *^b2^	5.37 ± 0.16 ^1^
	B	7.35 ± 0.20 *^c3^	5.82 ± 0.30 ^2^	5.31 ± 0.10 *^ab1^	5.24 ± 0.23 ^1^
	C	6.87 ± 0.12 ^ab4^	5.40 ± 0.19 ^3^	5.12 ± 0.16 ^a1^	5.39 ± 0.04 ^2^
	D	6.84 ± 0.18 ^a3^	5.66 ± 0.13 ^1^	5.21 ± 0.05 *^a2^	5.35 ± 0.04 *^2^

A (uninoculated control), B (inoculated with LAB 116), C (inoculated with LAB 31), and D (inoculated with LAB 116 and 31). Values are expressed as mean ± standard deviation. Means with different letters as superscripts (^a–c^) in the same column indicate significant differences (*p* ≤ 0.05) among batches from the same sampling day and maturation. Means with different numbers as superscripts (^1–4^) in the same row indicate significant differences (*p* ≤ 0.05) among sampling days of the same batch and elaboration. Means with an asterisk as a superscript (*) in the same column indicate significant differences (*p* ≤ 0.05) between elaborations (low and high relative humidity maturation) of the same batch and sampling day.

**Table 2:** Evolution of total viable counts (TVC), *Enterobacteriaceae* (EB), Gram-positive catalase-positive cocci (GC+), lactic acid bacteria (LAB), and yeasts from low and high relative humidity soft-ripened cheese elaborations.

Microbial Group	Batches	Days of Ripening			
		0	15	30	60
1. Low relative humidity maturation (log CFU/g)					
TVC	A	7.49 ± 0.09 *^c1^	9.68 ± 0.09 *^c3^	8.75 ± 0.15 ^de2^	8.85 ± 0.01 ^2^
	B	7.30 ± 0.06 *^bc1^	9.14 ± 0.04 ^a2^	7.58 ± 0.20 *^a1^	8.66 ± 0.91 ^12^
	C	6.58 ± 0.13 ^ab1^	9.05 ± 0.18 ^abc3^	7.92 ± 0.17 *^ab2^	8.22 ± 0.26 *^2^
	D	6.19 ± 0.13 *^a1^	9.26 ± 0.15 ^abc3^	8.19 ± 0.09 *^bc2^	8.81 ± 0.21 ^3^
	E	7.49 ± 0.09 *^c1^	9.53 ± 0.07 *^bc3^	9.06 ± 0.08 *^e2^	8.91 ± 0.04 *^2^
	F	7.30 ± 0.06 *^bc1^	8.78 ± 0.32 ^abc3^	7.85 ± 0.22 *^ab12^	7.94 ± 0.17 *^2^
	G	6.58 ± 0.13 ^ab1^	9.23 ± 0.04 *^ab3^	8.29 ± 0.19 *^bcd2^	8.03 ± 0.10 *^2^
	H	6.19 ± 0.13 *^a1^	9.21 ± 0.13 *^abc3^	8.17 ± 0.25 *^bc2^	8.23 ± 0.13 *^2^
EB	A	7.05 ± 0.08 *^c1^	8.65 ± 0.17 *^c2^	8.11 ± 0.03 *^c2^	7.01 ± 0.12 *^bc1^
	B	6.82 ± 0.12 *^bc12^	7.16 ± 0.03 *^ab2^	6.13 ± 0.98 *^a12^	6.97 ± 0.02 *^b1^
	C	6.01 ± 0.09 *^ab^	7.48 ± 0.05 *^abc^	7.01 ± 0.44 ^ab^	5.65 ± 0.79 ^abc^
	D	5.70 ± 0.04 ^a1^	7.64 ± 0.11 ^bc2^	7.38 ± 0.08 ^bc12^	6.01 ± 0.32 *^abc12^
	E	7.05 ± 0.08 *^c1^	8.56 ± 0.12 *^c4^	8.13 ± 0.09 *^c3^	7.28 ± 0.02 *^c2^
	F	6.82 ± 0.12 *^bc2^	7.11 ± 0.12 *^a2^	6.83 ± 0.22 *^ab2^	6.36 ± 0.07 ^a1^
	G	6.01 ± 0.09 *^ab^	7.36 ± 0.09 *^ab1^	7.23 ± 0.11 *^abc1^	6.26 ± 0.11 ^a1^
	H	5.70 ± 0.04 ^a1^	7.69 ± 0.23 ^bc3^	7.28 ± 0.08 *^abc23^	5.96 ± 0.47 ^abc12^
GC+	A	6.19 ± 0.16 *^c2^	5.52 ± 0.13 ^b1^	5.41 ± 0.30 ^c12^	6.29 ± 0.82 *^ab12^
	B	5.36 ± 0.13 ^b^	5.54 ± 0.18 *^b^	5.23 ± 0.21 ^c^	5.88 ± 0.47 *^ab^
	C	4.87 ± 0.04 *^a1^	4.52 ± 0.06 *^a2^	4.55 ± 0.28 ^ab12^	6.65 ± 0.24 *^b3^
	D	4.85 ± 0.13 ^a2^	4.67 ± 0.15 ^a12^	4.33 ± 0.08 ^a1^	6.12 ± 0.13 *^ab3^
	E	6.19 ± 0.16 *^c2^	5.29 ± 0.15 ^b1^	5.01 ± 0.09 ^bc1^	6.53 ± 0.41 *^ab12^
	F	5.36 ± 0.13 ^b^	5.37 ± 0.13 *^b^	5.36 ± 0.22 ^c^	6.50 ± 0.49 *^ab^
	G	4.87 ± 0.04 *^a2^	4.38 ± 0.08 ^a1^	4.80 ± 0.05 *^abc2^	5.29 ± 0.22 *^a3^
	H	4.85 ± 0.13 ^a1^	4.60 ± 0.18 ^a1^	4.67 ± 0.05 *^ab1^	5.71 ± 0.37 *^ab2^
LAB	A	7.12 ± 0.17 *^a1^	8.49 ± 0.00 *^bc2^	8.81 ± 0.06 *^b3^	8.84 ± 0.08 ^a23^
	B	7.56 ± 0.06 *^b1^	8.29 ± 0.03 ^ab2^	8.93 ± 0.20 ^a2^	9.37 ± 0.49 ^bc12^
	C	7.55 ± 0.03 ^b1^	8.31 ± 0.04 ^ab2^	8.89 ± 0.05 ^a3^	9.23 ± 0.02 *^c4^
	D	7.52 ± 0.03 *^ab1^	8.30 ± 0.10 ^ab2^	8.77 ± 0.07 ^ab3^	8.90 ± 0.06 *^ab3^
	E	7.12 ± 0.17 *^a1^	8.62 ± 0.11 *^c2^	8.96 ± 0.03 *^ab2^	8.82 ± 0.05 *^a2^
	F	7.56 ± 0.06 *^b1^	8.42 ± 0.12 *^bc2^	8.74 ± 0.06 *^ab3^	8.92 ± 0.07 *^ab3^
	G	7.55 ± 0.03 ^b1^	8.33 ± 0.11 ^ab2^	8.69 ± 0.03 ^a2^	9.08 ± 0.04 ^bc3^
	H	7.52 ± 0.03 *^ab1^	8.22 ± 0.06 ^a2^	8.97 ± 0.29 ^ab3^	8.98 ± 0.10 *^abc3^
Yeasts	A	7.29 ± 0.20 *^b1^	9.47 ± 0.20 *^cd3^	8.45 ± 0.10 ^c2^	8.90 ± 0.07 ^3^
	B	7.26 ± 0.20 *^b1^	9.11 ± 0.17 *^abc2^	7.29 ± 0.12 *^a1^	9.01 ± 0.15 ^2^
	C	6.27 ± 0.14 ^a1^	8.96 ± 0.18 *^ab3^	7.28 ± 0.50 *^a2^	9.11 ± 0.08 ^3^
	D	5.81 ± 0.23 ^a1^	9.21 ± 0.10 *^abcd3^	7.58 ± 0.16 *^a2^	8.22 ± 1.09 ^123^
	E	7.29 ± 0.20 *^b1^	9.48 ± 0.06 *^d4^	8.33 ± 0.12 *^b2^	8.74 ± 0.13 *^3^
	F	7.26 ± 0.20 ^b^	8.68 ± 0.24 ^a^	7.64 ± 0.36 *^abc^	8.75 ± 1.11
	G	6.27 ± 0.14 ^a1^	9.35 ± 0.13 ^bcd4^	7.65 ± 0.08 *^ab2^	8.97 ± 0.02 *^3^
	H	5.81 ± 0.23 ^a1^	9.42 ± 0.08 *^cd4^	7.71 ± 0.06 *^abc2^	9.05 ± 0.06 *^3^
2. High relative humidity maturation (log CFU/g)					
TVC	A	6.33 ± 0.02 *^a1^	9.42 ± 0.08 *^3^	8.65 ± 0.06 ^ab2^	9.03 ± 0.37^3^
	B	6.53 ± 0.06 *^c1^	8.90 ± 0.26^2^	8.96 ± 0.03 *^abc2^	9.18 ± 0.08^3^
	C	6.39 ± 0.07 ^ab1^	9.00 ± 0.16^2^	9.00 ± 0.03 *^bc2^	8.92 ± 0.29 *^2^
	D	6.48 ± 0.05 *^bc1^	9.17 ± 0.21^2^	8.99 ± 0.10 *^abc2^	9.14 ± 0.08^2^
	E	6.33 ± 0.02 *^aa1^	8.92 ± 0.05 *^3^	8.59 ± 0.04 *^a2^	9.15 ± 0.13 *^3^
	F	6.53 ± 0.06 *^c1^	8.77 ± 0.05 ^2^	8.99 ± 0.06 *^abc3^	9.27 ± 0.04 *^4^
	G	6.39 ± 0.07 ^ab1^	8.90 ± 0.17 *^2^	9.11 ± 0.05 *^c23^	9.36 ± 0.11 *^3^
	H	6.48 ± 0.05 *^bc1^	8.90 ± 0.07 *^2^	9.07 ± 0.06 *^c3^	9.52 ± 0.11 *^4^
EB	A	5.59 ± 0.07 *^1^	7.89 ± 0.39 *^2^	8.01 ± 0.05 *^b2^	4.92 ± 0.12 *^abcd1^
	B	5.69 ± 0.29 *^2^	7.83 ± 0.15 *^3^	7.76 ± 0.21 *^ab3^	3.30 ± 0.27 *^a1^
	C	5.71 ± 0.09 *^2^	7.85 ± 0.12 *^3^	7.45 ± 0.23 ^a3^	4.69 ± 0.48 ^abc1^
	D	5.57 ± 0.33 ^12^	7.22 ± 1.11 ^23^	7.51 ± 0.25 ^a3^	4.17 ± 0.69 *^ab1^
	E	5.59 ± 0.07 *^1^	7.71 ± 0.12 *^3^	7.55 ± 0.15 *^a3^	6.09 ± 0.24 *^cde2^
	F	5.69 ± 0.29 *^1^	7.90 ± 0.13 *^2^	7.78 ± 0.04 *^ab2^	6.24 ± 0.29 ^e1^
	G	5.71 ± 0.09 *^1^	7.86 ± 0.12 *^2^	7.75 ± 0.15 *^ab2^	6.16 ± 0.37 ^de1^
	H	5.57 ± 0.33 ^1^	7.56 ± 0.58 ^23^	7.79 ± 0.13 *^ab3^	5.51 ± 0.04 ^bcde12^
GC+	A	4.67 ± 0.26 *^a123^	5.48 ± 0.16^3^	5.11 ± 0.18 ^bc3^	4.23 ± 0.51 *^ab1^
	B	5.31 ± 0.11 ^b2^	5.18 ± 0.02 *^2^	5.13 ± 0.08 ^c2^	4.13 ± 0.08 *^ab1^
	C	4.59 ± 0.07 *^a2^	4.83 ± 0.07 *^2^	4.59 ± 0.07 ^abc2^	3.85 ± 0.09 *^a1^
	D	4.98 ± 0.22 ^ab2^	4.92 ± 0.16^2^	4.49 ± 0.15 ^a2^	3.85 ± 0.12 *^a1^
	E	4.67 ± 0.26 *^a^	5.31 ± 0.21	4.51 ± 0.50 ^a^	4.24 ± 0.56 *^a1^
	F	5.31 ± 0.11 ^ab^	5.01 ± 0.05 *	4.78 ± 0.40 ^abc^	4.85 ± 0.25 *^ab^
	G	4.59 ± 0.07 *^b^	4.44 ± 0.51	4.28 ± 0.10 *^ab^	3.89 ± 0.38 *^ab^
	H	4.98 ± 0.22 ^ab3^	4.81 ± 0.08^23^	4.23 ± 0.12 *^ab1^	4.48 ± 0.12 *^b12^
LAB	A	5.73 ± 0.08 *^a1^	8.06 ± 0.02 *^ab2^	7.83 ± 0.33 *^ab2^	8.43 ± 0.47 ^a2^
	B	7.68 ± 0.03 *^b1^	8.39 ± 0.11 ^bd2^	8.67 ± 0.11 ^bc3^	9.21 ± 0.15 ^bc4^
	C	7.62 ± 0.07 ^ab1^	8.46 ± 0.10 ^d2^	8.72 ± 0.09 ^c3^	8.84 ± 0.27 *^ab23^
	D	7.62 ± 0.02 *^ab1^	8.39 ± 0.05 ^d2^	8.54 ± 0.33 ^abc123^	9.14 ± 0.08 *^abc3^
	E	5.73 ± 0.08 *^a1^	7.76 ± 0.10 *^ac2^	7.58 ± 0.10 *^a2^	8.54 ± 0.21 *^a3^
	F	7.68 ± 0.03 *^b1^	8.17 ± 0.07 *^abcd2^	8.59 ± 0.06 *^abc3^	9.22 ± 0.03 *^bc4^
	G	7.62 ± 0.07 ^ab1^	8.03 ± 0.29 ^abcd1^	8.67 ± 0.14 ^c2^	9.20 ± 0.10 ^bc3^
	H	7.62 ± 0.02 *^ab1^	8.26 ± 0.03 ^cd2^	8.72 ± 0.12 ^c2^	9.37 ± 0.10 *^c3^
Yeasts	A	6.07 ± 0.13 *^1^	8.64 ± 0.07 *^b2^	8.58 ± 0.16 ^a2^	9.06 ± 0.18 ^ab3^
	B	6.32 ± 0.11 *^1^	8.35 ± 0.07 *^a2^	9.09 ± 0.14 *^c3^	9.08 ± 0.11 ^ab3^
	C	6.10 ± 0.07 ^1^	8.40 ± 0.10 *^a2^	8.89 ± 0.15 *^abc3^	8.90 ± 0.17 ^a3^
	D	6.25 ± 0.18 ^1^	8.40 ± 0.09 *^a2^	8.94 ± 0.01 *^bc3^	9.11 ± 0.08 ^ab3^
	E	6.07 ± 0.13 *^1^	8.68 ± 0.03 *^b2^	8.71 ± 0.12 *^ab2^	9.09 ± 0.11 *^ab3^
	F	6.32 ± 0.11 *^1^	8.44 ± 0.01 ^a2^	9.02 ± 0.08 *^bc3^	9.28 ± 0.12 ^b4^
	G	6.10 ± 0.07 ^1^	8.44 ± 0.02 ^a2^	8.92 ± 0.07 *^bc3^	9.35 ± 0.04 *^b4^
	H	6.25 ± 0.18 ^1^	8.25 ± 0.08 *^a2^	8.93 ± 0.10 *^bc3^	9.39 ± 0.14 *^b4^

A (uninoculated control), B (inoculated with LAB 116), C (inoculated with LAB 31), D (inoculated with LAB 116 and 31), E (inoculated with *L. monocytogenes*), F (inoculated with *L. monocytogenes* and LAB 116), G (inoculated with *L. monocytogenes* and LAB 31) and H (inoculated with *L. monocytogenes*, LAB 116 and 31). Values are expressed as mean ± standard deviation. Means with different letters as superscripts (^a–e^) in the same column indicate significant differences (*p* ≤ 0.05) among batches from the same sampling day and maturation. Means with different numbers as superscripts (^1–4^) in the same row indicate significant differences (*p* ≤ 0.05) among sampling days of the same batch and elaboration. Means with an asterisk as a superscript (*) in the same column indicate significant differences (*p* ≤ 0.05) between elaborations (low and high relative humidity maturation) of the same batch and sampling day.

**Table 3:** Evolution of *Listeria monocytogenes* counts (log CFU/g) for low and high relative humidity soft-ripened cheese elaborations at 0, 15, 30, and 60 days of ripening.

Batches	Days of Ripening			
	0	15	30	60
1. Low relative humidity maturation (log CFU/g)				
E	4.09 ± 0.15 *^ab1^	4.30 ± 0.27 *^b1^	5.73 ± 0.08 *^b2^	4.97 ± 0.45 ^b12^
F	4.05 ± 0.18 *^ab2^	3.75 ± 0.19 *^a12^	2.87 ± 0.04 *^a1^	3.61 ± 1.21 ^ab12^
G	4.16 ± 0.08 *^b3^	3.82 ± 0.05 *^a2^	2.88 ± 0.15 *^a1^	2.03 ± 0.69 ^a12^
H	3.89 ± 0.18 *^a3^	3.71 ± 0.15 *^a23^	2.69 ± 0.13 *^a1^	2.37 ± 0.22 ^a1^
2. High relative humidity maturation (log CFU/g)				
E	5.56 ± 0.10 *^2^	5.21 ± 0.20 *^b12^	4.58 ± 0.05 *^b1^	4.55 ± 0.05 ^b1^
F	5.42 ± 0.06 *^2^	4.46 ± 0.19 *^a1^	4.17 ± 0.26 *^ab1^	2.94 ± 1.14 ^ab12^
G	5.56 ± 0.08 *^3^	4.50 ± 0.02 *^ab2^	3.78 ± 0.33 *^ab23^	2.11 ± 0.57 ^ab1^
H	5.51 ± 0.05 *^4^	4.37 ± 0.09 *^a3^	3.46 ± 0.26 *^a2^	1.44 ± 0.83 ^a1^

E (inoculated with *L. monocytogenes*), F (inoculated with *L. monocytogenes* and LAB 116), G (inoculated with *L. monocytogenes* and LAB 31) and H (inoculated with *L. monocytogenes*, LAB 116 and 31). Values are expressed as mean ± standard deviation. Means with different letters as superscripts (^a,b^) in the same column indicate significant differences (*p* ≤ 0.05) among batches from the same sampling day and maturation. Means with different numbers as superscripts (^1–4^) in the same row indicate significant differences (*p* ≤ 0.05) among sampling days for the same batch and elaboration. Means with an asterisk as a superscript (*) in the same column indicate significant differences (*p* ≤ 0.05) between elaborations (low and high relative humidity maturation) of the same batch and sampling day.

## Data Availability

Data is contained within the article.
